# How Has COVID-19 Affected Mental Health and Lifestyle Behaviors after 2 Years? The Third Step of a Longitudinal Study of Italian Citizens

**DOI:** 10.3390/ijerph20010759

**Published:** 2022-12-31

**Authors:** Cristina Mazza, Eleonora Ricci, Marco Colasanti, Alessandra Cardinale, Francesca Bosco, Silvia Biondi, Renata Tambelli, Alberto Di Domenico, Maria Cristina Verrocchio, Paolo Roma

**Affiliations:** 1 Department of Neuroscience, Imaging and Clinical Sciences, “G. d’Annunzio” University of Chieti-Pescara, 66100 Chieti, Italy; 2 Department of Psychological, Health and Territorial Sciences, “G. d’Annunzio” University of Chieti-Pescara, 66100 Chieti, Italy; 3 Department of Human Neuroscience, Sapienza University of Rome, 00185 Rome, Italy; 4 Department of Dynamic and Clinical Psychology, and Health Studies, Sapienza University of Rome, 00185 Rome, Italy

**Keywords:** mental health, depression, anxiety, stress, follow-up, lockdown, pandemic, SARS-CoV-2, lifestyle changes, habits

## Abstract

The COVID-19 pandemic and its protective measures had a tremendous effect on the general population’s mental health and deeply affected their lifestyle. The present study carried out a longitudinal analysis to evaluate the long-lasting psychological effects of the pandemic and its impact on the general population’s day-to-day routine. Three points in time were considered: the initial period of the lockdown (T1; *n* = 2766; March 2020), the final period of the lockdown (T2; *n* = 439; May 2020) and two years after the lockdown (T3; *n* = 268; July 2022). Frequency analyses were carried out to examine which behavioral changes were maintained following the COVID-19 pandemic and lockdown; furthermore, a repeated measures ANOVA test was run to measure differences in depression, stress, and anxiety levels between the three periods considered; lastly, multivariable ordinal logistic regression analyses were carried out to examine which variables were associated with psychological distress more than two years after the lockdown. The results highlighted that depression at T3 was associated with depression at T2 and negative affect, whereas stress at T3 was associated with stress at T2 and detachment. The psychological effects and lifestyle changes are also discussed.

## 1. Introduction

The unexpected arrival and rapid spread of COVID-19 resulted in the WHO declaring a pandemic on 11 March 2020. Promptly, governments worldwide imposed stringent lockdowns and recommended health measures to contain the infection (e.g., social distancing and avoiding handshakes). Immediately, there was a concerted effort by the scientific communities, which vivisected the virus and studied its mechanisms of development, reproduction, and transmission. This joint effort led to the development of reliable vaccines in a very short period. The consequences of the virus have also not been overlooked by researchers, who have been concerned with investigating the effects of the disease not only on physical health, but also on mental health, along with the effect of the disease’s consequences (e.g., lockdowns and quarantine).

### 1.1. Psychological Effects

Many cross-sectional studies have been implemented to find out the immediate responses to the spread of the virus and the measures of contagion containment (for a review on the topic see [1]) in different countries. Among these, a study undertaken at the start of the outbreak in China [2] highlighted how 53.8% of participants (*n* = 1210) rated the psychological impact of the outbreak (thus, the self-perceived impact of the COVID-19 public health crisis on the citizens’ mental health) as moderate or severe, and 16.5%, 28.8% and 8.1% reported moderate to severe depressive, anxiety, and stress symptoms or levels, respectively. Also in Africa, a continent in which the outbreak of deadly and contagious diseases is not an uncommon event, high levels of psychological distress were found. Many indices of psychological distress (anxiety, stress, loneliness, and depression) during the outbreak of the pandemic were found to be very high in South Africa [3], and in a study performed in Nigeria it was highlighted that 23% of the participants (*n* = 502) experienced moderate to severe depression [4], while 25% experienced symptoms of severe posttraumatic-stress disorder. In New Zealand 30% of a demographically representative sample (*n* = 2010) reported moderate to severe psychological distress, and 16% reported moderate to high levels of anxiety [5]. In particular, the rates of psychological distress of those under 44 years of age were well above the country baseline levels. Moreover, it has been highlighted how both in New Zealand and Australia, two of the most important Digital Mental Health Services reported an increase of users greater than 100% following the pandemic outbreak [6]. In the United States, a study with a sample of adults without pre-existing mental health issues (*n* = 11.537) [7], highlighted that 39% and 19% reported anxiety and depressive symptoms, respectively, for at least three days during the week, which shows a high prevalence of psychological distress in this population as well. In Italy, one of the first studies after the outbreak of COVID-19 [8] showed that the prevalence of psychological distress was indeed higher than that in the baseline of the Italian population, with 32.4% (*n* = 2766) of participants having high and extremely high levels of depression, 18.7% with high and extremely high anxiety, and 27.2% with high and extremely high stress.

These studies, along with many others, aimed to explore the immediate effects of the pandemic, providing a snapshot of the entire world’s mental health conditions as well as those for specific populations [9,10]. Although all of these studies present at least a moderate impact of the pandemic on an individual’s mental health, the interpretation of the results in many cases suffers from a lack of comparison with the pre-COVID-19 levels of psychological distress. On the other hand, a more limited group of studies gives us information about the changes in mental health that may correspond to the pandemic’s development, studying the effects of the pandemic on mental health over time. An Italian longitudinal study, which collected the initial data set at the beginning of the first lockdown (March 2020) and performed a follow-up on the same participants (*n* = 439) in May 2020 [11], showed an increase in both stress and depression, but not in anxiety. Increases in depression were associated with higher levels of depression at the start of the lockdown and having fewer coping strategies, while higher stress levels were associated with having more stress at the start of the lockdown and being younger. In the US, a longitudinal study aimed at analyzing mental health differences in the general population between June 2020 and September 2020 [12], found that the prevalence of mental health symptoms stayed elevated, with no significant differences between the two times. In the UK, research comparing the prevalence of psychological distress in a representative probability-based sample (*n* = 10,657) [13], showed an increase of 5.8% between September 2020 (21.3%) and January 2021 (27.1%). This result highlights that the distress experienced by populations because of the pandemic continued to be present and indeed to increase even up to almost a year after its outbreak; thus, confirming the importance and the need for more longitudinal studies. Another British study [14] collected information on mental health and lifestyle behaviors in a sample of 160 adults, firstly in May–June 2020 and subsequently in May–June 2021. They found an increase in wellbeing and a reduction in anxiety levels, and that having children aged 12–17 was associated with an increased anxiety.

### 1.2. Lifestyle Changes

While the detrimental effects of the COVID-19 pandemic on mental health are well-documented, lifestyle changes remain under-researched. The population had, in fact, to adjust to the new challenges posed by the pandemic and its related preventive and protective measures. This impacted people’s day-to-day routines and behaviors, some of which are adaptive (e.g., hand washing), and their maintenance should be promoted, and some of which are maladaptive (e.g., social avoidance) and could impair an individual’s quality of life or degenerate into medical and psychological conditions. International studies have reported various lifestyle changes during the pandemic, focusing on sleep, nutrition, and exercise. While the pandemic affected everyone in different ways, as evidenced, for instance, by the fact that both an increased consumption of healthy and unhealthy food was reported [15], its overall effects on the population were mostly negative. In fact, one of the most consistent results was a negative effect on the quality of sleep [16,17,18]; indeed, a meta-analysis [19] on sleep problems during the COVID-19 outbreak indicated that approximately 40% of the general and healthcare populations were affected. Similarly, most studies investigating dietary habits [17,20] reported an increase in food consumption and weight gain. Additionally, a systematic review [21] highlighted that, in many cases, changes in physical activity involved a decrease in exercising and an increase in sedentary behavior. Most of the follow-up studies conducted to verify whether these changes would be maintained covered a very short time frame; therefore, it is still uncertain whether these behavioral changes will be long-lasting. Theoretical perspectives, such as the Health Belief Model, propose that the implementation and maintenance of new health behaviors are linked to the perception of that situation as severe or life-threatening. This is corroborated, for instance, by the observation that smokers who undergo invasive medical treatments are more likely to quit smoking than smokers who are medically managed [22]. Similarly, considering negative behavioral responses, healthcare workers that tended SARS patients, when surveyed from 13 to 25 months after the SARS outbreak, reported reducing face-to-face patient contact, decreased work hours, increased smoking and alcohol consumption, as well as more frequent sick absences [23]. Furthermore, [24] found that during the MERS outbreak, people exhibited hospital avoidance behaviors, as suggested by a 17.2% reduction of the number of outpatient visits in the first two months after the outbreak. Therefore, it could be concluded that the behaviors developed during the COVID-19 outbreak might be maintained even in the post-pandemic period. 

### 1.3. Research Aims

The present study aimed to provide a further snapshot of the psychological impact of COVID-19, after more than two years of its spread, on the same Italian citizens already surveyed in March and May 2020. As the previously published longitudinal studies have collected data relating to two time-points, to the best of our knowledge, this is the first longitudinal research based on three time-steps. Furthermore, we sought to investigate the changes in the major lifestyle habits of daily life for the Italians interviewed.

## 2. Materials and Methods

### 2.1. Procedures

We used an anonymous online questionnaire to track the psychological response of the Italian citizens to the COVID-19 blockade, more than 2 years after its inception. The survey was administered cross-sectionally on the same online platform used for the earlier stages of this longitudinal study [8,11]. The link was disseminated to participants who had consented to the first survey to be contacted for follow-up and had provided their email addresses for this purpose. Data were collected from 30 June to 11 July 2022, using a survey covering sociodemographic and COVID-19-related information, mental health symptoms, and lifestyle habits. Ethical approval was obtained from the Institutional Committee of the Department of Human Neuroscience, Faculty of Medicine and Dentistry, ‘Sapienza’ University of Rome (IRB-2020-6), by the principles contained in the Declaration of Helsinki.

### 2.2. Participants

**Figure 1:**
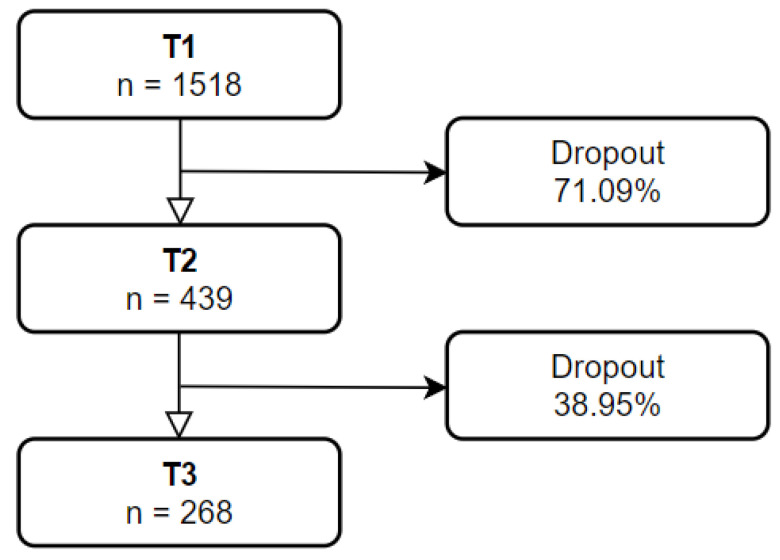
Flowchart of the participants’ attrition.

In the first national survey (T1) [8], 1518 respondents (out of 2766) expressed their willingness to be contacted for a follow-up study and they were invited to participate via email. Among them, 439 took part in the first follow-up study (T2): the dropout rate from time T1 to T2 was 71.09%. Among them, 279 agreed to be surveyed for the third step (T3). Eleven participants (out of 279) were excluded for registering a double set of answers, from which only one set was retained. Thus, the final sample comprised 268 participants (the dropout rate from time T2 to T3 was 38.95%), with 55 males (20.5%) and 213 (79.5%) females. Figure 1 highlights the participants’ attrition. The average age was 37.68 years (SD = 12.91; range 21–73). More descriptive statistics are presented in the Supplementary Material (Table S1). All participants voluntarily responded to the anonymous survey and indicated their informed consent within. The procedures were clearly explained, and participants could interrupt or quit the survey at any point without explaining their reasons for doing so.

### 2.3. Follow-Up Measures

*Sociodemographic data and COVID-19 related information.* Sociodemographic data were collected with regards to biological sex, age, education, marital and parental status, employment status and income, region of residence, and any history of stressful situations and medical problems related to COVID-19 or not. It was also asked what containment measures, introduced in March 2020 to prevent the spread of COVID-19, were still taken (e.g., “avoid hugs with people, even when they do not appear to have flu symptoms?”; “Avoid handshakes with people, even when they do not appear to have flu symptoms?”).

*Lifestyle habits.* Further information was collected regarding the acquired or lost lifestyle habits because of COVID-19. Participants were asked whether their habits regarding smoking, sleep, physical activity, nutrition, alcohol, relationships with family members, social life, public transportation usage, online shopping, social networking, and remote working had changed after the spread of COVID-19 (e.g., “Compared to before the pandemic, from when COVID-19 spread to now, how would you rate your drinking habits?”). 

*Psychological impact and mental health*. Mental health was measured using the Depression, Anxiety, and Stress Scale—21 items (DASS-21)—Italian adaptation [25]. The DASS-21 is a set of three self-report scales designed to measure the emotional states of depression, anxiety, and stress. Each of the three scales contains seven items, divided into subscales with a similar content. Items 3, 5, 10, 13, 16, 17, and 21 comprise the Depression subscale (e.g., “In the last 7 days, I couldn’t seem to experience any positive feeling at all”; “In the last 7 days, I found it difficult to work up the initiative to do things”); items 2, 4, 7, 9, 15, 19, and 20 comprise the Anxiety subscale (e.g., “In the last 7 days, I experienced trembling”; “In the last 7 days, I was worried about situations in which I might panic and make a fool of myself”); and items 1, 6, 8, 11, 12, 14, and 18 comprise the Stress subscale (e.g., “In the last 7 days, I tended to over-react to situations”; “In the last 7 days, I felt that I was using a lot of nervous energy”). All subscales are rated on a 4-point Likert scale ranging from 0 (never) to 3 (almost always). The DASS-21 obtained high reliability in the Italian validation study, with Cronbach’s alphas of 0.74, 0.82, and 0.85 for the Anxiety, Depression, and Stress subscales, respectively; Cronbach’s alpha for the total scales was 0.90. In our sample, Cronbach’s alphas were 0.92, 0.82, and 0.92 for the Depression, Anxiety, and Stress subscales, respectively; Cronbach’s alpha for the total scales was 0.95.

### 2.4. Statistical Analysis

The statistical analysis was performed using SPSS v.28 (IBM Corp, Armonk, NY, USA) [26]. The absolute and percentage frequencies of the lifestyle changes were computed. A repeated-measures ANOVA test was performed on the DASS-21 subscale scores to measure differences in stress, anxiety, and depression levels between the three periods considered [i.e., March 2020 (T1), May 2020 (T2), and July 2022 (T3)]. In the event of significance of the repeated-measures ANOVA, post-hoc tests were conducted with the Bonferroni adjustment. The significance level was set at 0.05. Subsequently, multivariable ordinal logistic regression analyses were performed to investigate the association between DASS-21 subscales’ scores after two years of COVID-19 spread and the following independent variables: the DASS-21 subscales’ scores from the initial period of the lockdown and during the final period of the lockdown, and the significant factors from the first period of the lockdown and from the final period of the lockdown [8,11]. The collinearity assumption was checked before running the model. The analysis was performed using a stepwise variable selection (with the threshold level of statistical significance for each variable to enter the model set to *p* < 0.05).

## 3. Results

### 3.1. Lifestyle Changes

The descriptive statistics relative to lifestyle changes are reported in Table 1. In the Supplementary Material (Figure S1) a graphical representation of the prevailing modes of the lifestyle variables is reported.

### 3.2. Differences in Depression, Anxiety, and Stress Levels

**Figure 2:**
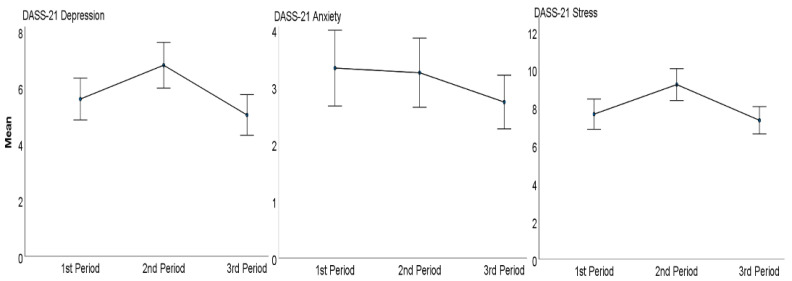
Descriptive plots comparing depression, anxiety, and stress levels, as measured with the DASS-21, between the three periods considered (*n* = 268).

Table 2 reports the average scores on the DASS-21 subscales pertaining to the three periods considered. A graphical representation is provided in Figure 2. The results of the repeated measures ANOVA revealed a difference in both the Depression and the Stress subscale scores (see Table 2).

To summarize, on the Depression subscale the post hoc tests with the Bonferroni adjustment showed that there was a statistically significant difference between periods T1 and T2 (Difference_1,2_ = −1.20; *p* < 0.001) and between periods T2 and T3 (Difference_2,3_ = 1.77; *p* < 0.001), but not between periods T1 and T3 (Difference_1,3_ = 0.57; *p* = 0.445). 

The same pattern was found on the Stress subscale. The post hoc tests with the Bonferroni adjustment revealed a statistically significant difference between periods T1 and T2 (Difference_1,2_ = −1.56; *p* < 0.001) and between periods T2 and T3 (Difference_2,3_ = 1.88; *p* < 0.001), but not between periods T1 and T3 (Difference_1,3_ = 0.32; *p* > 0.999) (see Figure 2).

### 3.3. Regression Analysis

The descriptive statistics of the community sample reported by Bottesi et al. [25] showed that the DASS-21 scores for depression and stress were classified into three ranges: medium, high, and extremely high, setting the cut-offs at one and two standard deviations from the average, to establish the average and high levels, respectively. Multivariate ordinal logistic regression models were then constructed to capture associations between levels of depression and stress, as measured by the DASS-21, and the sociodemographic variables along with the personality traits. The variables statistically significant in Mazza et al. [8] and Roma et al. [11] were chosen, adding the values of depression and stress measured at T2. Only the statistically significant variables are reported, as the stepwise technique was implemented. Regarding the DASS-21 anxiety variable, no regression was performed, as no statistically significant differences were obtained between the three measurement times.

#### 3.3.1. Depression

The results for depression levels showed that 73.4% (*n* = 127) of respondents had an average level, 20.2% (*n* = 35) were in the high range, and 6.4% (*n* = 11) were in the extremely high range. The prediction model for depression showed that the final model was better than the one with only the intercept to our observed data (χ^2^ (2) = 37.832, *p* < 0.001). The test of parallel lines was not significant (χ^2^ (2) = 3.881, *p* = 0.144). Nagelkerke’s pseudo R^2^ of 0.294 indicated that the significant variables explained approximately 29.4% of the variability. Higher levels of the PID-5-BF Negative Affect and the DASS-21 Depression at T2 were significantly associated with higher levels of depression at T3 (see Table 3).

#### 3.3.2. Stress

The results for stress levels showed that 67.1% (*n* = 116) of respondents had an average level, 28.9% (*n* = 50) were in the high range, and 4.0% (*n* = 7) were in the extremely high range. The prediction model for stress showed that the final model was better than the one with only the intercept to our observed data (χ^2^ (2) = 36.492, *p* < 0.001). The test of parallel lines was not significant (χ^2^ (2) = 0.739, *p* = 0.691). Nagelkerke’s pseudo R^2^ of 0.281 indicated that the significant variables explained approximately 28.1% of the variability. Higher levels of the PID-5-BF Detachment and DASS-21 Stress at T2 were significantly associated with higher levels of stress at T3 (see Table 4).

## 4. Discussion

Over these nearly 3 years, the COVID-19 pandemic has radically changed people’s lives with consequences of physical and mental health and behavioral changes in everyday life. This research represents the third step of a nationwide longitudinal study on the Italian population that aimed at assessing the psychological distress and the lifestyle behavior changes almost three years after the COVID-19 pandemic onset. The first two steps of this longitudinal study, conducted during the Italian lockdown [8] and at the end of the Italian lockdown [11], have highlighted that the Italian population experienced high levels of psychological distress during the lockdown period and very high levels of psychological distress at the end of the lockdown period in May 2020. Overall, the findings of the present study show that most of the participants are now experiencing average levels of depression, stress, and anxiety, whilst a decreased percentage of people is experiencing high and extremely high levels of distress, compared to the first two periods [8,11]. Not surprisingly, the unprecedented situation and the uncertainty about what the pandemic would entail caused high and extremely high levels of depression, stress, and anxiety as assessed during and at the end of the lockdown in Italy, compared to those measured approximately three years later. This decrement trend is clear especially for the anxiety levels over the three periods considered, even if not to a statistically significant degree. Indeed, compared to anxiety levels measured during the COVID-19 lockdown (T1; M = 3.33) and then at the end of the lockdown (T2; M = 3.25), participants assessed in T3 reported a further decrease in anxiety levels (M = 2.73). This result is not startling as anxiety levels at T1 might be explained by the uncertainty of the threat represented by COVID-19, which had not yet been fully realized; and the uncertainty itself could have exacerbated subthreshold anxiety symptoms within the population. Nonetheless, it is possible to assume that as the knowledge about the virus has increased (i.e., its spread and related symptoms), and as the adaptation to protective measures and also the prompt response with the vaccination campaigns has increased, so have the anxiety levels steadily decreased over the three-year pandemic period. Furthermore, this finding on the decrement in anxiety levels confirms the results from other longitudinal studies performed on the UK population [13,14]. Conversely, depression and stress levels did not follow this decreasing linear trend. High and extremely high levels of depression and stress were reported by most participants during the COVID-19 lockdown (T1; [8]), with a further increment of depression and stress levels at the end of the lockdown in Italy (T2; [11]) and a decrement in the third period (T3). Indeed, in the present study, for both depression and stress levels, statistically significant differences were found between T1 and T2 and between the T2 and T3, but no significant differences were found between periods T1 and T3, even though both depression and stress levels at T3 are lower compared to those at T1. The peak of depression and stress levels registered at the end of the Italian lockdown is not surprising. Indeed, studies on forced cohabitation during lockdowns have demonstrated that long periods of lockdown can have a huge impact on mental health, therefore, reducing well-being [1]. Moreover, the government-mandated measures to manage the infection have exposed individuals to new living situations (i.e., physical distancing and remote working, etc.), with significant consequences for important life domains (e.g., work and interpersonal relationships). The return to “normality”, with the lightening of the government-mandated measures, has contributed to the decrement of depression and stress levels (T3), which are even lower than those at the beginning of lockdown (T1), but still higher compared to the Italian normative population [25]. This highlights that the COVID-19 pandemic is still affecting people’s mental health nearly 3 years after the virus outbreak.

Among the factors previously found to be significantly associated with levels of depression, higher levels of depression during the final period of lockdown (T2) and higher levels of the personality domain, negative affect, were identified as significant predictors of higher levels of depression at T3. These results concur with the first two steps of this longitudinal study [8,11]. Indeed, higher levels of depression at the beginning of the lockdown were significantly associated with further increased levels at the end of the lockdown, and higher levels of depression at T2 were found to be significantly associated with higher levels of depression at T3. Furthermore, as widely reported by the literature, the personality domain of negative affect has been identified as a good index of internalizing psychopathology (e.g., [28,29,30]). 

Higher levels of stress during the final period of lockdown (T2) and higher levels of detachment were significantly associated with higher levels of stress in the present study. This finding mirrors those of the first two studies [8,11]. Similar to the results for depression levels, the higher levels of stress at the beginning of the lockdown were significantly associated with further increased levels at the end of the lockdown, and higher levels of stress at T2 were found to be significantly associated with high levels of stress in the present study. Moreover, the relation between detachment and stress levels is consistent with the findings of previous studies, which highlighted that internalizing maladaptive personality traits (e.g., detachment, negative affectivity, and psychoticism) might influence subjective sensitivity in the experience of psychological distress and are strongly associated with psychological distress [31]. Finally, the role of pathological personality domains in an individual’s psychological reactions to COVID-19 has been further examined in a recent research study [32,33], which confirmed their association with negative mental health outcomes during the current pandemic.

Behavioral changes considered in the present study can be loosely divided into two main categories: on one side, lifestyle changes affecting various facets of a person’s life; on the other, health behaviors related to the infection-containment measures (e.g., avoiding handshakes or wearing a face mask). Regarding the former, diet, exercise, sleep, and relationships can be considered the pillars of lifestyle [34]. Most participants in the present survey reported no changes in food intake (56%), diet (48.2%), and alcohol consumption (68.7%); among those reporting changes, most participants disclosed eating less food (60.7%), eating more healthily (67.2%), and drinking less alcohol (55.1%). These results seem to deviate from what has been suggested in the international literature: systematic reviews indicate both a decrease in diet quality and an increase in alcohol consumption [35,36,37]. It could be argued that most of the studies considered refer to the period during the lockdown, in which people may have adopted unhealthy dietary behaviors (e.g., snacking); however, a systematic review of longitudinal studies similarly reported a decreased adherence to healthy diets and increased alcohol consumption [38]. A possible explanation for these findings is the observation that, in Mediterranean countries, adherence to the Mediterranean diet, typically considered healthy, has increased during the pandemic [39]. The results for physical activity show that 33.6% of participants reported no changes in their exercising habits. Among those who reported changes, 23.3% stopped exercising, 32% exercised less, 18% started exercising, and 26.7% exercised more. These results paint a heterogeneous picture, reflecting the importance of individual variables such as age [40] and gender [41] in influencing changes in physical activity. Interestingly, participants reported that compared to the pre-pandemic period they exercised more at home and outdoors (11.9% pre-pandemic vs. 20.9 post-pandemic and 17.9% pre-pandemic vs. 24.6% post-pandemic, respectively) and less at the gym or in predisposed facilities (44.6% pre-pandemic vs. 20.1% post-pandemic). This could reflect the preference for exercising in less crowded and more aerated spaces. The results for sleep show that most participants reported no changes in the number of hours spent sleeping (56.7%) and its quality (49.3%). Among those reporting changes, 62% reported sleeping for fewer hours and 88% reported a deteriorated quality of sleep. These results concur with the international literature, which consistently indicates an increase in the prevalence of sleeping disorders during the COVID-19 pandemic [19]. Finally, the results for social interactions show that most participants reported no change in the quality of family relationships (53%); among those reporting changes, half felt that the quality of their relationships with family members had deteriorated whereas the other half felt it had improved. Moreover, only a portion of the sample reported the absence of change in the frequency of family relating (39.6%) and social time (17.2%). Indeed, the majority reported changes which were related to the specifically decreased frequency of family relating (68.4%) and decreased social time (78.6%), highlighting the presence of social withdrawal in a large segment of this study’s sample.

The second category of changes relates to health behavior being adopted or ceased during the pandemic. Most of the participants in the sample in the present study maintained the recommended protective behaviors (coughing and sneezing in a handkerchief or elbow, 78%; washing or disinfecting hands often when outside, 77.2%; wearing facial masks in enclosed spaces, 60.8%; avoiding crowded places, 57.1%; avoiding touching the face with the hands, 54.1%). Other protective behaviors were maintained by a relatively smaller portion of the sample, especially those related to physical contact and proximity (avoiding hugs, 35.8%; avoiding handshakes, 42.9%; maintaining at least a 1 m distance from others, 38.4%). Overall, these changes could have been maintained because of widespread concerns regarding not only oneself, with 30.2% of the participants reporting that they were between being somewhat and very concerned about their own health, but were predominantly concerned about their loved ones’ health: where 54.9% of the participants reported being between somewhat and very concerned about the health of loved ones.

## 5. Strengths and Limitations

The present research conducted a longitudinal study to examine the effects of the COVID-19 pandemic on the mental health of the general population considering three key moments (the initial period of the lockdown—March 2020, the period right after the initial lockdown—May 2020, and two years after the initial lockdown—July 2022). To the best of our knowledge, this is the first nationwide longitudinal research based on three time-steps. This study has, however, some limitations. First, the survey measure relied on voluntary sampling, which might not be representative of the general population, being composed for the most part of females (89.5%) and individuals with higher levels of education (78% having at least graduated). Furthermore, this study was characterized by a high attrition rate (81.62% from T1 to T3). Both these limitations might have a common origin based on indications from the literature that females have higher response rates to surveys, compared to men, and they tend to be more cooperative [42,43,44]; similarly, other sociodemographic variables, such as a low educational level, impact the risk of non-response and dropout [45]. Lastly, the psychological constructs examined relied exclusively on self-reports implemented via the Internet and were not assessed with other methodologies (e.g., semi-structured interviews) for convergent validity. Despite these limitations, the present study highlights that the negative mental health consequences following the COVID-19 pandemic and the related protective measures are still present in the general population, and indicates which variables are associated with a more adverse psychological outcome.

## 6. Conclusions

The results of the present research underline the need to systematically address the mental health fallout from the pandemic. The Italian government, on this matter, has opened applications to claim a “psychologist bonus” that can be used to cover some of the psychological treatment expenses incurred by those struggling with their mental health following the COVID-19 outbreak. This commendable initiative in addressing the population’s post-pandemic recovery is hopefully the first step in a larger preventive and protective mental health program that also addresses those individuals who were specifically affected by the pandemic. The results of the present study provide data supporting the need for further initiatives to protect mental health.

## Figures and Tables

**Table 1:** Descriptive statistics of the lifestyle changes of the sample (*n* = 268).

		Group	*N* (%)
Smoking		No changes	207 (77.2%)
		I stopped smoking	18 (6.7%)
		On average I smoke fewer cigarettes per day	17 (6.3%)
		I started smoking	8 (3.0%)
		On average I smoke more cigarettes per day	15 (5.6%)
Sleep	Number of hours spent sleeping	No changes	152 (56.7%)
		I dedicate fewer hours to sleep	70 (26.1%)
		I dedicate more hours to sleep	43 (16.0%)
	Quality of hours spent sleeping	No changes	132 (49.3%)
		I consider sleep quality worsened	117 (49.7%)
		I consider the quality of sleep to have improved	16 (6.0%)
Physical/sporting activity		No changes	90 (33.6%)
		I have stopped engaging in physical activity/sport	40 (14.9%)
		I spend less time on physical activity/sports	55 (20.5%)
		I have started physical activity/sport	31 (11.6%)
		I spend more time on physical activity/sport	46 (17.2%)
	Where you carried out physical/sporting	Did not engage in physical/sporting activity	62 (23.1%)
		Predominantly at home	32 (11.9%)
		Predominantly in the gym/swimming pool/prepared facilities	120 (44.8%)
		Mostly outdoors	48 (17.9%)
	Where you carry out physical/sporting now	Did not engage in physical/sporting activity	86 (32.1%)
		Predominantly at home	56 (20.9%)
		Predominantly in the gym/swimming pool/prepared facilities	54 (20.1%)
		Mostly outdoors	66 (24.6%)
Food	Average daily food intake	No changes	150 (56.0%)
		I take in less food	68 (25.4%)
		I consume more food	44 (16.4%)
	Diet	No changes	129 (48.2%)
		I feel that I eat less healthily	43 (16.0%)
		I feel that I eat more healthily	88 (32.8%)
Alcohol		No changes	184 (68.7%)
		I have stopped consuming alcohol	6 (2.2%)
		I consume less alcohol	43 (16.0%)
		I have started consuming alcohol	3 (1.1%)
		I consume more alcohol	26 (9.7%)
Social Relations	Frequency of relations with family members	No changes	106 (39.6%)
		Decreased the frequency of dealings	104 (38.8%)
		Increased the frequency of dealings	48 (17.9%)
	Quality of relationships with family members	No changes	142 (53.0%)
		Worsened the quality of relations	58 (21.6%)
		Improved the quality of relations	58 (21.6%)
	Social time (e.g., aperitifs, concerts)	No changes	46 (17.2%)
		I have discontinued participation	22 (8.2%)
		I have decreased participation	165 (61.6%)
		I started to participate	3 (1.1%)
		I increased participation	20 (7.5%)
Public Transport		No changes	135 (50.4%)
		I have stopped using public transport	47 (17.5%)
		On average I use public transport less	64 (23.9%)
		I have started to use public transport	3 (1.1%)
		On average I use public transport more	5 (1.9%)
	Quality of life as a result of changes in the use of public transport (only for those who have had changes)	Worse	12 (10.1%)
		Slightly worsened	33 (27.7%)
		Neither worsened nor improved	48 (40.3%)
		Slightly improved	11 (9.2%)
		Improved	13 (10.9%)
Online shopping		No changes	91 (34.9%)
		I have stopped buying online	1 (0.4%)
		On average I shop online less	13 (4.9%)
		I have started to shop online	21 (7.8%)
		On average I shop online more	125 (46.6%)
	What you buy most online:		
	1. I do not shop online	Yes	25 (9.3%)
		No	243 (90.7%)
	2. Food	Yes	23 (8.6%)
		No	245 (91.4%)
	3. Clothing	Yes	94 (35.1%)
		No	174 (64.9%)
	4. Cosmetics	Yes	39 (14.6%)
		No	229 (85.4%)
	5. Games and video game	Yes	16 (6.0%)
		No	252 (94.0%)
	6. Furniture and home accessories	Yes	44 (16.4%)
		No	224 (83.6%)
	7. Various	Yes	122 (45.5%)
		No	146 (54.5%)
	Quality of life as a result of changes in the habits of online shopping (only for those who have had changes)	Worse	2 (1.3%)
		Slightly worsened	20 (12.5%)
		Neither worsened nor improved	73 (45.6%)
		Slightly improved	47 (29.4%)
		Improved	18 (11.3%)
Social networks	Using social networks to stay in touch with contact with people	No changes	107 (39.9%)
		I do not use social networks	29 (10.8%)
		On average I use social networks less	17 (6.3%)
		I started using social networks	18 (6.7%)
		On average I use social networks more	79 (29.5%)
	Quality of life as a result of changes in the habit of using social networks to communicate (only for those who have had changes)	Worse	16 (11.2%)
		Slightly worsened	32 (22.4%)
		Neither worsened nor improved	62 (43.4%)
		Slightly improved	23 (16.1%)
		Improved	10 (7.0%)
Remote working		No changes	26 (9.7%)
		I do not work in remote working	124 (46.3%)
		On average I work less in remote working	4 (1.5%)
		I started working in remote working	52 (19.4%)
		On average I work more in remote working	43 (16.0%)
	Quality of life following the novelty of remote working (only for those who have had changes)	Worse	10 (4.5%)
		Slightly worsened	18 (8.1%)
		Neither worsened nor improved	108 (48.4%)
		Slightly improved	41 (18.4%)
		Improved	43 (19.3%)
Health	Concerned about your health	Not at all	33 (12.3%)
		Little	65 (24.3%)
		Neither concerned neither not	66 (24.6%)
		Somewhat	58 (21.6%)
		Very	23 (8.6%)
	Concerned about the health of loved ones	Not at all	16 (6.0%)
		Little	40 (14.9%)
		Neither concerned neither not	42 (15.7%)
		Somewhat	90 (33.6%)
		Very	57 (21.3%)
Economic stability	Concerned about his own economic stability	Not at all	34 (12.7%)
		Little	40 (14.9%)
		Neither concerned neither not	61 (22.8%)
		Somewhat	56 (20.9%)
		Very	54 (20.1%)
How much more vulnerable/fragile he/she perceives his/her existence		Not at all	24 (9.0%)
		Little	28 (10.4%)
		Neither vulnerable neither not	51 (19.0%)
		Somewhat	92 (34.3%)
		Very	50 (18.7%)

Note. In the analysis, the missing data were also considered, unless otherwise specified.

**Table 2:** The difference between the three periods in levels of stress, anxiety, and depression, as measured with the DASS-21 (*n* = 268).

DASS-21 Subscales	*M* (*SD*)	*F*	*p*-Value	*η^2^_P_*
DASS-21 Depression *		*F*_(2,171)_ = 13.279	<0.001	0.072 (medium)
	T1: 5.60 (4.95) T2: 6.80 (5.41) T3: 5.03 (4.81)			
DASS-21 Anxiety		*F*_(2,171)_ = 2.436	0.089	0.014
	T1: 3.33 (4.41) T2: 3.25 (4.04) T3: 2.73 (3.14)			
DASS-21 Stress *		*F*_(2,171)_ = 14.212	<0.001	0.076 (medium)
	T1: 7.65 (5.32) T2: 9.21 (5.61) T3: 7.33 (4.81)			

Note. Statistically significant effects (*p* < 0.01) are marked (*). The final column reports the effect size (partial eta squared, *η*^2^*_P_*). Concerning magnitude, *η*^2^*_P_* = 0.01 was considered indicative of a small effect, *η^2^_P_* = 0.06 of a medium effect, and *η*^2^*_P_* = 0.13 of a large effect [27].

**Table 3:** The association between significant variables (*n* = 173 *) and levels of depression at T3.

Predictor	Estimate	EXP (B)	95% C.I.		*p*
Negative Affect	0.216	1.241	0.037	0.396	0.018
DASS-21 Depression at T2	0.172	1.188	0.082	0.262	<0.001

Note. * The sample size is due to the missing data.

**Table 4:** The association between significant sociodemographic variables (*n* = 173 *) and levels of stress at T3.

Predictor	Estimate	EXP (B)	95% C.I.		*p*
Detachment	0.212	1.236	0.046	0.377	0.012
DASS-21 Stress at T2	0.181	1.198	0.092	0.271	<0.001

Note. * The sample size is due to the missing data.

## Data Availability

The datasets used and/or analyzed during the current study are available from the corresponding author on reasonable request.

## Supplementary Materials

The following supporting information can be downloaded at: https://www.mdpi.com/article/10.3390/ijerph20010759/s1, Figure S1: Prevailing Modes of Lifestyle Variables; Table S1: Descriptive Statistics of the Sample (*N* = 268), Table S2: Containment Measures Introduced in March 2020 and Still Retained.
