# Supplementary material for: How Has COVID-19 Affected Mental Health and Lifestyle Behaviors after 2 Years? The Third Step of a Longitudinal Study of Italian Citizens
Source: Int J Environ Res Public Health. 2022 Dec 31;20(1):759. doi: 10.3390/ijerph20010759 (PMC9819689; doi:10.3390/ijerph20010759)
Supplement: Supplementary file 1 [file ijerph-20-00759-s001.zip › ijerph-2100733-supplementary.pdf]

Supplementary Material

Table S1. Descriptive Statistics of the Sample (*N* = 268).

**Characteristic**	**Group**	***N* (%)**
Gender	Female	213 (89.5%)
	Male	55 (20.5%)
Age		
M (SD)	37.68 (12.91)	268 (100%)
Min–Max	21–73	
Education	Primary school	1 (0.4%)
	Middle school diploma	4 (1.5%)
	High school diploma	62 (23.1%)
	Graduate	127 (47.4%)
	Postgraduate	74 (27.6%)
Occupation	Employee	133 (49.6%)
	Freelancer	56 (20.9%)
	Unemployed	26 (9.7%)
	Student	47 (17.5%)
	Retired	6 (2.2%)
Income	0 €	50 (18.7%)
	<10,000 €	44 (16.4%)
	<15,000 €	0 (0%)
	<26,000 €	0 (0%)
	<55,000 €	0 (0%)
	<75,000 €	46 (17.2%)
	<120,000 €	60 (22.4%)
	≥120,000 €	68 (25.4%)
Marital status	Unmarried	79 (29.5%)
	Married	67 (25.0%)
	In a relationship	110 (41.0%)
	Separated/divorced	9 (3.4%)
	Widower	3 (1.1%)
Child(ren)	Yes	92 (34.3%
	No	176 (65.7%)
if yes:		
M (SD)	1.69 (0.65)	88 (95.7%)
Min–Max	1–4	
Citizenship	Italian	267 (99.6%)
	Foreign	1 (0.4%)
Region of residence	North	61 (22.8%)
	Center	141 (52.6%)
	South	66 (24.6%)
COVID-19–independent trauma in the past year	Diagnosis of disease	3 (1.1%)
	Dismissal/loss of job	48 (17.9%)
	Death of a family member	162 (60.4%)
	Legal problems	4 (1.5%)
	Separation or divorce	19 (7.1%)
	Moving city or country	6 (2.2%)
	None of the above	26 (9.7%)
COVID-19–dependent trauma in the past year	Diagnosis of disease	2 (0.7%)
	Dismissal/loss of job	12 (4.5%)
	Death of a family member	224 (83.6%)
	Legal problems	3 (1.1%)
	Separation or divorce	3 (1.1%)
	Moving city or country	7 (2.6%)

	None of the above	17 (6.3%)
Medical issues:		
No pathology	Yes	175 (63.3%)
	No	93 (34.7%)
Cardiovascular pathology	Yes	12 (4.5%)
	No	256 (95.5%)
Respiratory pathology	Yes	9 (3.4%)
	No	259 (96.6%)
Oncological pathology	Yes	14 (5.2%)
	No	254 (94.8%)
Orthopedic pathology	Yes	17 (6.3%)
	No	251 (93.7%)
Psychiatric pathology	Yes	6 (2.2%)
	No	262 (97.8%)
Other pathology	Yes	49 (18.3%)
	No	219 (81.7%)
Psychotherapy	Yes	93 (34.7%)
	No	175 (63.3%)
Psycopharmaceuticals	Yes	25 (9.3%)
	No	243 (90.7%)

Table S2. Containment Measures Introduced in March 2020 and Still Retained.

Which of the Infection Containment Measures Introduced in March 2020 to Prevent the Spread of COVID-19 Do You Still Follow?	Group	*N* (%)
Avoid hugs	Yes	96 (35.8%)
	No	156 (56.0%)
Avoid handshakes	Yes	115 (42.9%)
	No	131 (48.9%)
Maintain a distance of at least 1 meter from others	Yes	103 (38.4%)
	No	143 (53.4%)
Avoid places where there are crowds	Yes	153 (57.1%)
	No	93 (34.7%)
Wash (or disinfect with alcohol-based gel) hands often outside the home	Yes	207 (77.2%)
	No	39 (14.6%)
Avoid touching face with hands	Yes	145 (54.1%)
	No	101 (37.7%)
Sneeze or cough using a handkerchief or elbow	Yes	209 (78.0%)
	No	37 (13.8%)
Use masks in enclosed spaces	Yes	163 (60.8%)
	No	83 (31.0%)

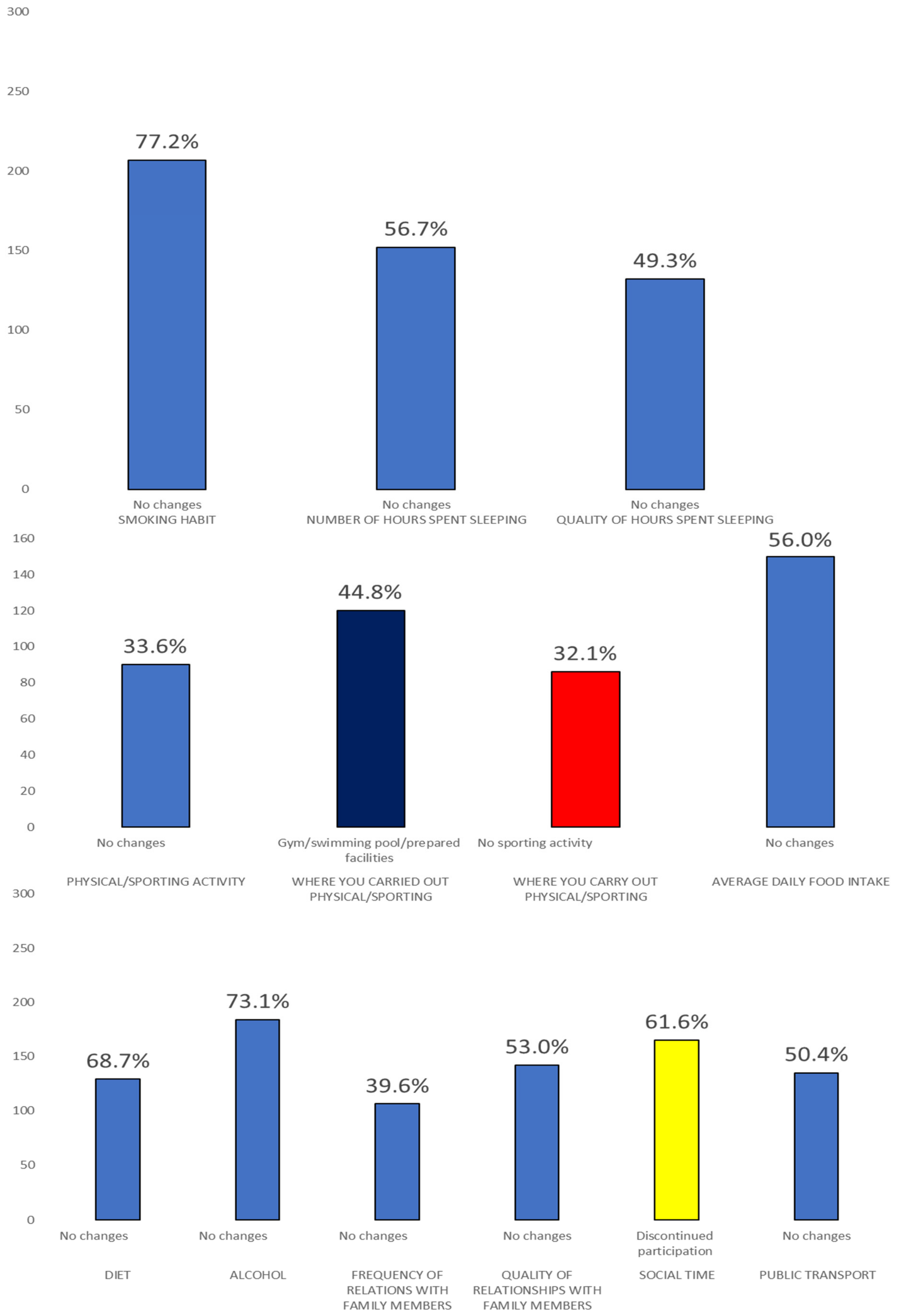
300
250
200
150
100
50
0
77.2%
56.7%
49.3%
No changes
SMOKING HABIT
No changes
NUMBER OF HOURS SPENT SLEEPING
No changes
QUALITY OF HOURS SPENT SLEEPING
160
140
120
100
80
60
40
20
0
33.6%
44.8%
32.1%
56.0%
No changes
Gym/swimming pool/prepared facilities
No sporting activity
No changes
PHYSICAL/SPORTING ACTIVITY
WHERE YOU CARRIED OUT PHYSICAL/SPORTING
WHERE YOU CARRY OUT PHYSICAL/SPORTING
AVERAGE DAILY FOOD INTAKE
300
250
200
150
100
50
0
68.7%
73.1%
39.6%
53.0%
61.6%
50.4%
No changes
No changes
No changes
No changes
Discontinued participation
No changes
DIET
ALCOHOL
FREQUENCY OF RELATIONS WITH FAMILY MEMBERS
QUALITY OF RELATIONSHIPS WITH FAMILY MEMBERS
SOCIAL TIME
PUBLIC TRANSPORT

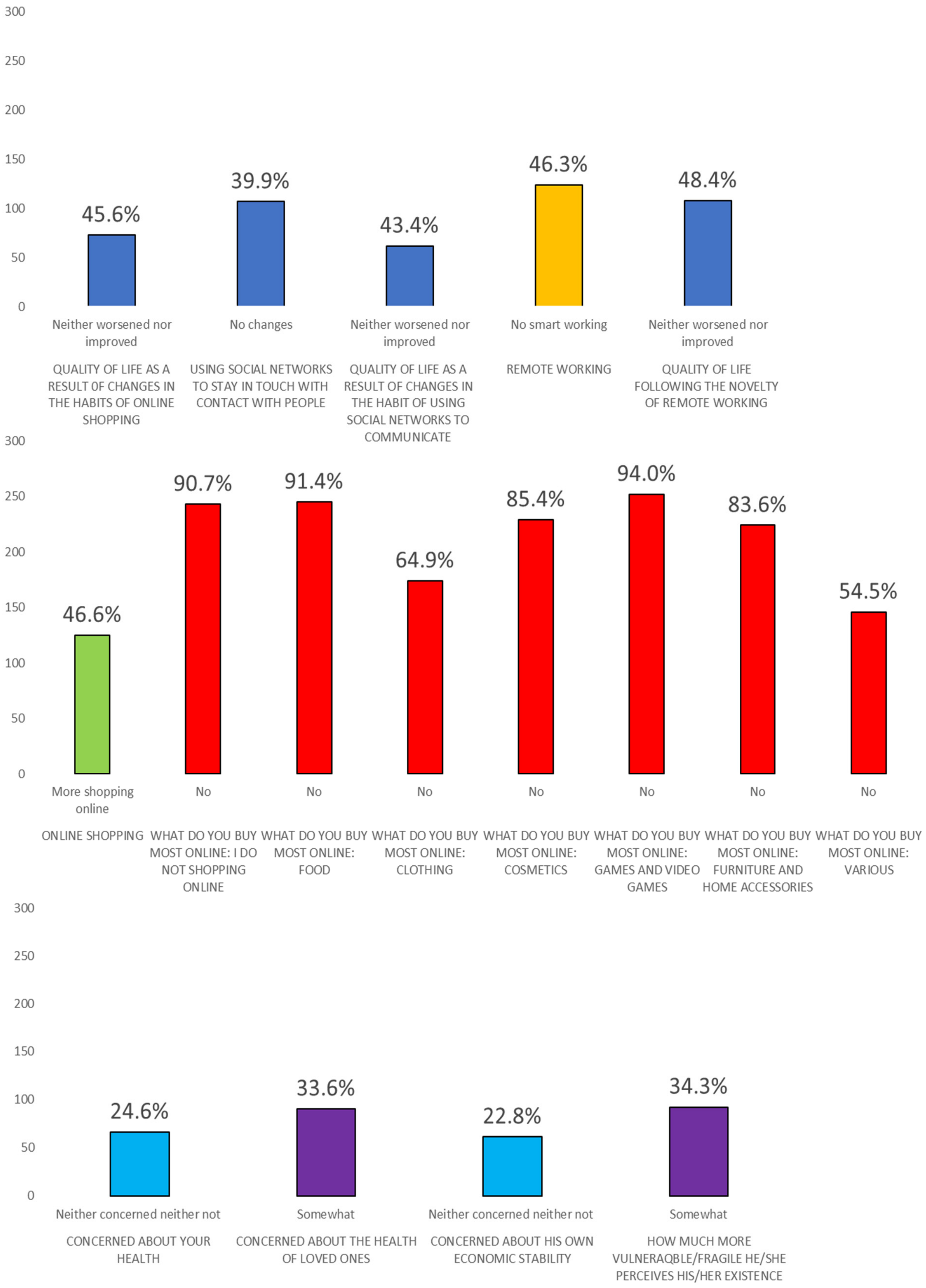

Figure S1. Prevailing Modes of Lifestyle Variables.
